# Spontaneous radial artery pseudoaneurysm in an infant due to idiopathic medial hypoplasia – a case report

**DOI:** 10.1080/23320885.2019.1614449

**Published:** 2019-06-17

**Authors:** William W. Kesler, Joseph J. Maleszewski, Alexander H. Payatakes

**Affiliations:** a Department of Radiology, Penn State Health Milton S. Hershey Medical Center, Hershey, PA, USA;; b Department of Laboratory Medicine and Pathology, Mayo Clinic, Rochester, MN, USA;; c Department of Orthopaedics and Rehabilitation, Penn State Health Milton S. Hershey Medical Center, Hershey, PA, USA

## Abstract

Spontaneous pseudoaneurysms are uncommon vascular lesions in children. A seven-month-old boy presented for management of a painless, pulsatile mass of the volar forearm identified as a pseudoaneurysm of the radial artery on imaging, definitively treated with surgical excision. Histology was concerning for fibromuscular dysplasia, without additional lesions on whole-body imaging.

## Introduction

Aneurysms are uncommon vascular lesions in the paediatric population. Defined as expansion of the vascular contour beyond its expected boundaries, they typically present as expanding pulsatile masses, occasionally causing pain and/or compression of adjacent structures. They are categorised into true aneurysms or pseudoaneurysms based on the constituents of the aneurysmal wall. True aneurysms are expansions involving all three vascular layers and are typically associated with connective tissue disease, trauma, or arterial infection [1]. Pseudoaneurysms are characterised by areas of medial defect, typically traumatic, contained by surrounding adventitia [2]. The mainstay of treatment for upper extremity aneurysms remains prompt surgical resection, due to the risks of rupture and thromboembolic events distal to the lesions [1,3].

Herein, we present a rare case of a seven-month-old boy with a spontaneous pseudoaneurysm of the radial artery and severe idiopathic medial hypoplasia, most consistent with fibromuscular dysplasia, definitively treated with surgical excision.

## Case report

A seven-month-old boy was referred for evaluation of a painless mass of the left wrist. His parents noticed a soft tissue mass 4–5 mm in diameter four months earlier with progressive enlargement. He had a benign birth history and no history of trauma, vascular access, or inherited vascular, collagen, or rheumatologic disorders.

**Figure 1.**
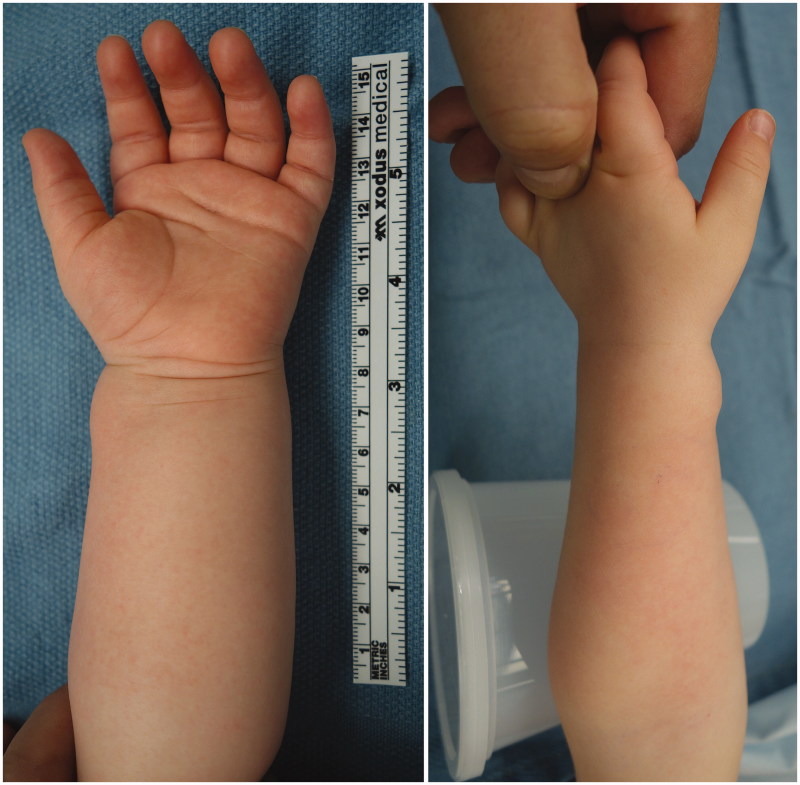
On initial presentation, the patient had a 1 cm slightly bluish, pulsatile, non-tender soft tissue mass over the course of the left radial artery, concerning for a vascular anomaly.

**Figure 2.**
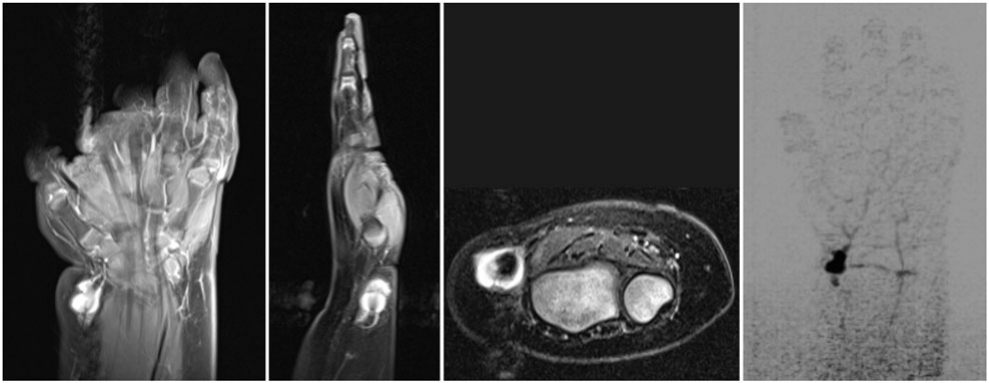
Magnetic resonance angiography (coronal, sagittal, and axial views) revealed a 1.1 × 0.8 × 1.0 cm mass with retrograde arterial filling arising laterally off the distal radial artery from the superficial palmar arch with evidence of thrombosis of the artery proximal to the lesion and intact superficial and deep palmar arches.

On examination, the patient had a 1 cm bluish, non-tender, pulsatile mass over the left radial artery (Figure 1). A Doppler-assisted Allen test raised concerns regarding the patency of the involved artery. Arterial duplex revealed a 9 × 9 mm suspected true aneurysm with normal flow distally. Magnetic resonance angiography (MRA) demonstrated a 1.1 × 0.8 × 1.0 cm mass arising off the radial artery with retrograde filling from the superficial palmar arch and thrombosis proximal to the lesion (Figure 2). Surgical excision with possible reconstruction was recommended.

**Figure 3.**
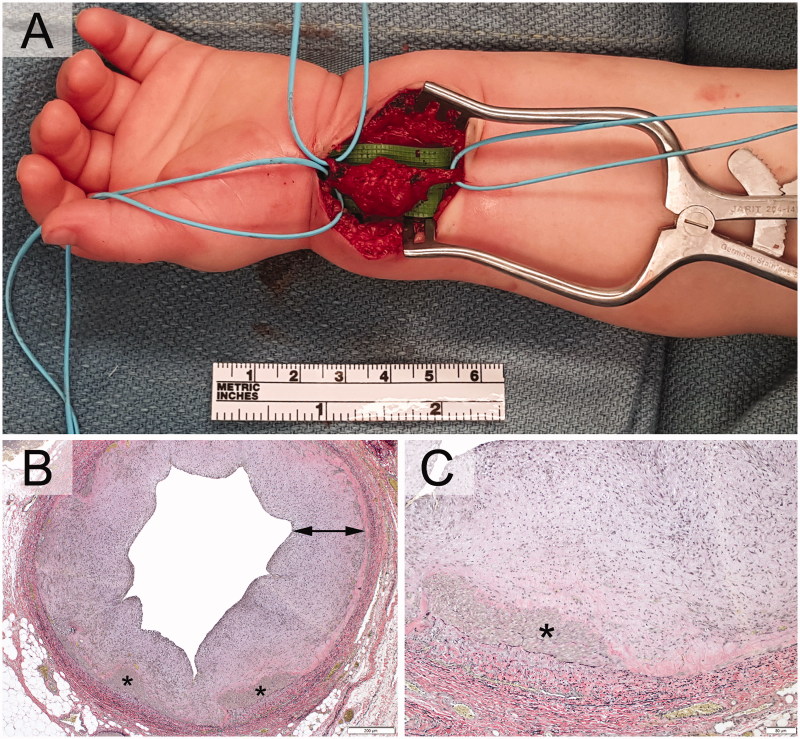
A. Intraoperatively, the lesion was pulsatile, grossly multi-lobular, and reddish and focally bluish in colour with fibrous attachments to the flexor carpi radialis sheath. The radial artery was resected proximally to the mid-forearm and distally just past the wrist flexion crease. Photomicrographs exhibiting pseudoaneurysmal radial artery with scant residual media (asterisks) and striking intimal fibroplasia (arrow). Verhoeff-van Giesson stains (3B) original magnification, ×40. (3C) original magnification, ×500.

Intraoperatively, the arterial lesion was noted to be multi-lobular, focally bluish, and adhered to the flexor carpi radialis sheath, with the volar carpal branch of the radial artery exiting the mass. (Figure 3(A)). The radial artery was dissected to healthy vessel wall (proximally to the mid-forearm and distally past the wrist flexion crease). Trial clamping of the radial artery proximally and distally demonstrated maintenance of brisk distal capillary refill throughout, confirming a complete, patent arch. The radial artery was transected and the abnormal segment excised. Robust arterial backflow was noted from the distal stump of the radial artery following transection. Reconstruction with vein graft was not felt to be necessary and would additionally result in significantly prolonged anaesthesia time for such a young patient. The radial artery was therefore simply ligated.

On histopathology, the specimen exhibited medial attenuation with large areas of complete media loss, consistent with pseudoaneurysm (Figure 3(B,C)). Also noted was intimal fibroplasia with incorporating mural thrombus. These changes were noted to be most consistent with fibromuscular dysplasia of the medial type. The differential diagnosis included heritable connective tissue disorders such as type IV Ehlers-Danlos (EDS), Loeys-Dietz, and Marfan syndromes.

Given the abnormal pathology and young presentation age, the patient underwent a genetics workup to evaluate for possible connective tissue disorders. He was noted to have a large body habitus and hyperelastic skin, concerning for EDS. MRA exams of the carotids, brain, chest, abdomen, and pelvis, recommended by vascular surgery at the age of thirteen months to assess for any further large/medium vessel lesions, were unremarkable. A cardiovascular genetics workup of sixteen genes associated with EDS, Marfan, and familial thoracic aortic aneurysm and dissection (FTAAD) found no pathogenic variants.

At three years postoperatively, the patient used both hands symmetrically. There was no clinical evidence of growth arrest or cold intolerance. Ongoing monitoring for additional vascular abnormalities is planned.

## Discussion

Pseudoaneurysm is an exceedingly rare diagnosis in children and is typically the result of traumatic vascular access, a history of which was present in both previously reported infantile brachial artery pseudoaneurysms [4,5]. No standard imaging workup exists, but options include ultrasound, computed tomography angiography (CTA), MRA, and traditional angiography. Historically, the treatment for peripheral pseudoaneurysms has been surgical excision with ligation, repair, or reconstruction of the involved artery [1,4,5]. For upper extremity pseudoaneurysms and aneurysms, the decision to reconstruct the resected segment is based on intraoperative inadequacy of the perfusion of the hand. Physeal arrest has been reported after radial and femoral arterial cannulations complicated by thromboembolic events but has not been reported after ligation or repair of the arterial structures [6]. Less invasive treatment strategies have been reported, including external compression devices, thrombin injection of the adventitia, coil embolisation, and stenting [2,6,7]. The finding of a spontaneous radial artery pseudoaneurysm in an infant without corresponding vascular trauma is rare and, to our knowledge, has not been reported in the literature.

Fibromuscular dysplasia (FMD) is an occlusive disease of medium-sized arteries characterised by medial fibroplasia, disruption of the elastic membrane, and formation of saccular aneurysms, classically in a “string of beads” pattern [8]. It is believed to be an inherited disorder with autosomal dominant transmission of variable penetrance [9]. FMD characteristically involves the renal and carotid arteries, with reports of the brachial, radial, and ulnar arterial involvement [9, 10]. Three types have been described: intimal, perimedial, and medial, the latter being the most common [9]. Upper extremity FMD lesions have been treated with surgical excision and angioplasty. Critical ischaemia of the hand requiring arterial reconstruction has been reported due to embolisation of both forearm arteries in an elderly patient [11]. Infantile-onset FMD has been reported as the cause of ischaemic strokes in children as young as eight months [12]. Current genetic testing cannot exclude FMD, which is likely a heterogeneous disease with no known associated genes. In our case, although no “string of beads” was evident on imaging, the singular pseudoaneurysm histologically resembled medial FMD and may have represented an early stage of such a lesion. To this point, our patient has demonstrated no other signs of vasculopathy.

Ehlers-Danlos syndrome is a group of inherited connective tissue disorders presenting with problems related to collagen synthesis and processing. Classically, patients have hyperelastic skin, joint hypermobility, multiple ecchymoses, and subcutaneous pseudotumors [13]. Six types are currently recognised, with rare subtypes found in individual families. Type IV (Sack-Barabas) is the predominantly vascular form, with autosomal dominant and recessive variants. The genetic defect in *COL3A1* results in decreased or absent production of type III collagen, which is vital for vessel wall integrity and platelet plug formation. Patients bruise easily, and pseudoaneurysms of the subclavian, axillary, radial arteries may occur after minor trauma or angiography. Spontaneous ruptures of the aorta, major visceral arteries, and peripheral arteries in this population have also been reported [13]. Surgery is reserved for patients with life-threatening conditions [13]. Marfan syndrome, the most common inherited connective tissue disorder, occurs due to an autosomal dominant mutation in the FBFN1 (fibrillin 1) gene. Classically, patients have long bones and often arachnodactyly, dural ectasia, aortic root disease, or mitral prolapse. Spontaneous radial artery aneurysms have been reported in patients with this disorder [14]. Loeys-Dietz syndrome is an autosomal dominant disorder involving a mutation in the transforming growth factor-beta (TGF-β) pathway. Five types have been identified; patients often have hypertelorism and V-shaped uvulae. Aortic root dissection is present in 98% of patients, with generalised arterial tortuosity and peripheral aneurysms (brachial, carotid) present in 92% [15].

In summary, we presented a rare case of a 7-month-old boy with a spontaneous radial artery pseudoaneurysm and severe idiopathic medial hypoplasia, most consistent with fibromuscular dysplasia, treated definitively with surgical excision of the lesion. Angiographic workup demonstrated no additional lesions, and genetic analysis did not provide a definitive diagnosis. No medium-term sequelae have been encountered, and the patient has continued to have full use of the operative extremity. Monitoring for additional vascular abnormalities is ongoing, given the theoretical risk of early-onset stroke.
